# The Emerging Role of Voltage-Gated Sodium Channels in Tumor Biology

**DOI:** 10.3389/fonc.2019.00124

**Published:** 2019-03-06

**Authors:** Weijia Mao, Jie Zhang, Heinrich Körner, Yong Jiang, Songcheng Ying

**Affiliations:** ^1^Key Laboratory of Oral Disease Research of Anhui Province, College & Hospital of Stomatology, Anhui Medical University, Hefei, China; ^2^Department of Immunology, School of Basic Medical Sciences, Anhui Medical University, Hefei, China; ^3^Key Laboratory of Anti-inflammatory and Immunopharmacology, Institute of Clinical Pharmacology, Anhui Medical University, Hefei, China; ^4^Menzies Institute for Medical Research, University of Tasmania, Hobart, TAS, Australia

**Keywords:** VGSCs, tumors, metastasis, expression regulation, mechanism

## Abstract

Voltage-gated sodium channels (VGSCs) are transmembrane proteins which function as gates that control the flux of ions across the cell membrane. They are key ion channels for action potentials in excitable tissues and have important physiological functions. Abnormal function of VGSCs will lead to dysfunction of the body and trigger a variety of diseases. Various studies have demonstrated the participation of VGSCs in the progression of different tumors, such as prostate cancer, cervical cancer, breast cancer, and others, linking VGSC to the invasive capacity of tumor cells. However, it is still unclear whether the VGSC regulate the malignant biological behavior of tumors. Therefore, this paper systematically addresses the latest research progress on VGSCs subunits and tumors and the underlying mechanisms, and it summarizes the potential of VGSCs subunits to serve as potential targets for tumor diagnosis and treatment.

## Introduction

In general, ion channels including VGSC, are hydrophilic protein microchannels traversing the semi-permeable cell membranes regulating intracellular ion concentrations, facilitating signaling pathways, and influencing cell behavior. VGSC play a key role in generating electrochemical action potentials in excitable cells, participate in maintaining homeostasis and are an important components of physiological activities such as muscle contraction, cell proliferation (1) and cognitive activities. Interestingly, VGSCs are also expressed in “non-excitable” cells such as fibroblasts, various immune cells of the myeloid linage (2), and glial cells such as astrocytes, microglia, and oligodendrocyte precursor cells (3). An exciting example is the expression of the VGSC NaV1.5 in late endosomes of macrophages (4). After activation with LPS this sodium channel regulates phagocytosis and endosomal pH causing acidification via sodium efflux. Furthermore, these molecules are also present in cancer cells from e.g., colon, breast, prostate, and non-small cell lung cancer. In these transformed cells they are involved in tumor growth, invasion, and metastasis (5–9). This contribution of VGSC to cancer malignancy and to the resistance of tumors to chemotherapy drugs has become the focus of research. Nevertheless, the specific underlying mechanisms of Na^+^ channels affecting these changes in cancer cells are still unclear and need to be explored further.

## Structure And Function OF VGSCs

**Figure 1:**
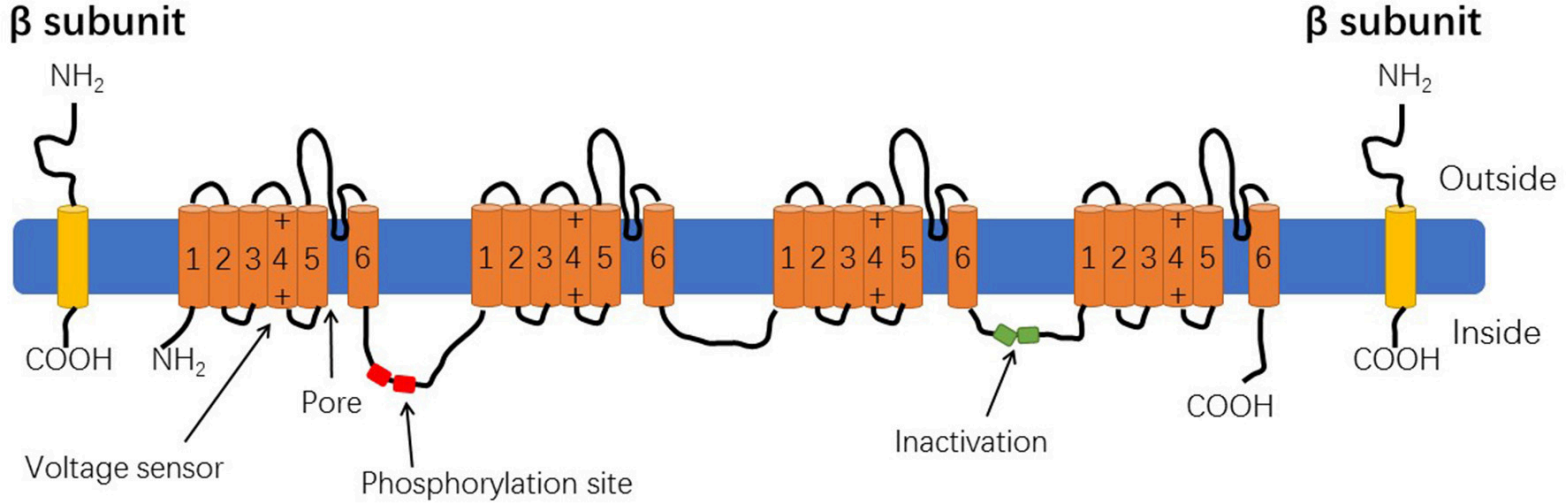
The structure of voltage-gated sodium channel.

VGSC is a multi-subunit, transmembrane glycoprotein that is activated by voltage changes in the cell membrane. It's usually composed of a α subunit (220–260 kD) and one or more β subunits (33–36 kD) (Figure 1) (10). The nine α subunits are termed Nav1.1–Nav1.9 which are encoded by nine different genes (*SCN1A-SCN11A*). Tetrodotoxin is a specific blocker of sodium channels and according to their varying sensitivity to tetrodotoxin VGSCs are divided into tetrodotoxin-sensitive (TTX-S) and tetrodotoxin-resistance (TTX-R) forms (11). Nanomolar concentrations of TTX can block TTX-sensitive VGSC function, whereas resistant forms are blocked by micromolar concentrations of TTX. TTX-resistant molecules include Nav1.5, Nav1.8, and Nav1.9. All other α subunits are TTX-S (12). The α subunit comprises the functional centers of VGSC and consists of 4 highly similar transmembrane domains (I-IV) (Figure 1). Each domain contains 6 transmembrane segments of an α-helix (S1–S6). S4 is a voltage sensor which, after stimulation, moves the S4 transmembrane segment out of the membrane, causing the three-dimensional structure of the channel protein to change, thereby achieving channel opening (13). The S5 and S6 sections enclose the central pore domain and their insertion sequences constitute the selectivity filter, which determines Na^+^ selectivity and functions as a gate (14). There are multiple protein kinase phosphorylation sites between the I and II domains. The short chains of the III and IV domains in the cytoplasmic region, which can close open channels from inside of the cell during rapid depolarization, causing channel inactivation. This area could be an important target for drugs (10). In addition, studies also have found that there are three hydrophobic amino acids (1488-IFM−1490) in the middle of the polypeptide linkage site between S6 in the III domain and S1 in the IV domain that can inactive channels (15). The traditional idea is that a single α-subunit acts as a monomer, but it has been proposed in the literature that the sodium channel α subunit not only physically interact with each other, but they actually combine, and act as dimers (16).

The β subunit is an auxiliary subunit of the Na+ channel and has five subtypes: β1, β1B, β2, β3, and β4, which are encoded by the genes *SCN1B-SCN4B*. With the exception of β1B, the β subunit consists of an α-helical transmembrane domain linking the extracellular N-terminus and the intracellular C-terminus. The β1B subunit variant lacks a transmembrane protein domain with a secretory function. The β1 and β3 subunits have high homology, bind to the α subunit in a non-covalent manner, and the β2 and β4 subunits have high homology, and are bound by a covalent disulfide bond. The β subunit does not participate in the formation of the pore structure of the channel, but changes the gating, voltage dependence, and kinetics of the VGSC alpha subunit, thereby regulating the excitability of cells *in vivo* (17). In addition, a recent study has found that β subunits can affect the function of the α subunit and regulate its biophysical and pharmacological properties along with channel phosphorylation (18). Furthermore, the β subunit regulates the expression of voltage-gated ion channels on the plasma membrane and acts as a cell adhesion molecule that interacts with cytoskeletal proteins, extracellular matrix, and other cell adhesion molecules affecting cell adhesion and migration activities (19, 20).

The α subunit contains four transmembrane domains (I–IV), each domain contains 6 transmembrane helices (S1–S6), and S4 is a voltage sensor. Phosphorylation sites of multiple protein kinases between the I and II domains, intracellular short peptides between the III and IV domains can inactivate the channel. The β subunits contain an extracellular immunoglobulin loop, intracellular C-terminal domain, and a transmembrane domain.

It is of interest that the expression of VGSC in glial cells such as astrocytes results in fully functional channels that can support a significant action potential (21). This suggests an as of yet unresolved role of this class of molecules in astrocytes. In microglia it has been shown that sodium channels are regulating, for example, cell behavior such as lamellipodia formation and motility (22) and specifically the production and release of the proinflammatory cytokines IL-1α, IL1β, and TNF-α (23).

### VGSCs and Tumors

Previous analyses of various tumor tissues have found that their intrinsic concentration of sodium ions is significantly higher than that of surrounding normal tissues and is closely related to the formation of tumors (24). Furthermore, it could be shown that a high expression of VGSC in various tumors is closely related to malignant biological behavior.

#### Prostate Cancer

Using the patch clamp technique to analyse sodium currents in rat prostate cancer cells an inward sodium current could be detected in highly metastatic Mat-Ly-Lu but not in weakly metastatic AT-2 prostate cancer cells. This flow of sodium ions could be blocked by 600 nM tetrodotoxin. Moreover, results from the study showed that the use of TTX significantly reduced the invasive ability of Mat-Ly-Lu cells while it had no significant effect on AT-2 cells (25). Furthermore, a comprehensive quantitative expression study of all subtypes of the VGSC α subunit was carried out in normal prostate, benign prostatic hyperplasia (BPH) and prostate cancer cells. It found that in PC-3 and LNCaP cells, two typical prostate cancer cell lines, Nav1.6 and Nav1.7 were highly expressed with their mRNA levels 6–27 times higher in PC-3 and LNCaP than in normal or BPH samples. However, while the expression of Nav1.6 and Nav1.7 is highly functional in highly metastatic PC-3 cells it has no effect in LNCaP cells as shown by using patch clamp technique to record whole cell currents (26). In the Copenhagen rat model of prostate cancer the number of lung metastases is reduced by >40% after blocking VGSC activity in primary tumors using tetrodotoxin. This extends the lifespan of the experimental animals greatly suggesting that VGSC activity promotes prostate cancer metastasis *in vivo* (27). Furthermore, a recent study found that Naringenin, a natural compound found in citrus fruits and tomatoes, reduced the invasion and proliferation of prostate cancer MAT-LyLu cells by inhibiting the activity of VGSCs encoded by the *SCN9A* gene (28).

#### Breast Cancer

Breast cancer is the most common malignant cancer among women. Its mortality is extremely high and death occurs mostly after metastasis. Roger et al. reported for the first time that a unique, rapid inward sodium current was found in highly invasive breast cancer MDA-MB-231 cells, whereas no introversion current was found in weakly invasive MCF-7 and MDA-MB-468 cells. In addition, currents in MDA-MB-231 cells were blocked by high concentrations of tetrodotoxin (TTX). This reduced invasiveness by ~30% (29). Similarly, Gillet et al. demonstrated that TTX blocked continuous sodium influx in MDA-MB-231 breast cancer cells, and knockdown of NaV1.5 expression reduced cell invasiveness, while using sodium channel activator veratridine had the opposite effects (30). In a separate investigation it could be shown that the activity of Na^+^ channels increased cell motility and endocytosis. As a consequence, this caused an increased invasiveness of metastatic human breast cancer cells. In addition, the expression of a “neonatal” splicing form of VGSC in these cells was increased and directly related to metastatic potential (31). Recent studies have found that the protein levels of Nav1.5 are upregulated in metastatic breast cancer cells compared to normal breast tissue. Moreover, down-regulation of Nav1.5 significantly decreased tumor growth, invasion, and spread of metastases to liver, lung, and spleen (8).

Phenytoin, an antiepileptic drug, has been found to inhibit the transient and sustained Na(+) flow recorded in strongly metastasizing MDA-MB-231 breast cancer cells (32). It inhibited the migration and invasion of MDA-MB-231 cells significantly at concentrations within the therapeutic range for treating epilepsy, but had no effect on weakly metastatic MCF-7 cells that did not display Na+ currents. Thus, phenytoin may be a potential therapeutic drug to treat patients with metastatic breast cancer. In addition, the clinically used drug Ranolazine could inhibit Nav1.5 current in breast cancer cells and reduce the invasiveness of cancer cells *in vitro*. In *in vivo* experiments, mice injected with Ranolazine significantly reduced lung colonization by breast cancer cells expressing Nav1.5 (33). Furthermore, a recent study designed and synthesized 5 small compounds as inhibitors of Nav1.5-associated inward currents in MDA-MB-231 breast cancer cells. The most active compound blocked the sodium flow by 34.9 ± 6.6% at 1 μM and inhibited MDA-MB-231 cell invasion without affecting cell viability (34).

#### Cervical Cancer

The whole-cell patch clamp technique was employed to isolate and identify voltage-gated Na+ currents as a major component of the inward flow in cervical cancer cells (35). While in normal cervical biopsies exclusively the expression Nav1.4 transcripts was detectable by RT-PCR in primary cultures obtained from human cervical cancer biopsies, the mRNAs of the additional subunits Nav1.2, Nav1.6, and Nav1.7 could be detected. This study showed for the first time the functional and distinct expression of specific VGSCs human cervical cancer cells, suggesting that the VGSCs potentially could be used as a marker for diagnosis or treatment prognosis of human cervical cancer. Another study showed that the mRNA level of Nav1.6 α subunit in cervical cancer samples was ~40-fold higher than in non-cancerous cervical biopsies (6). Using cell patch clamp experiments, the Nav1.6-specific toxin Cn2 (*Centruroides noxius* Beta-mammal toxin) blocked ~30% of the total sodium current confirming the functional activity of Nav1.6 channels in the plasma membrane of cervical cancer cells. *In vitro* assays, the blocking of VGSC by tetrodotoxin and Cn2 did not affect cell proliferation and migration, but reduced the invasiveness of primary cervical cancer cells by about 20%. This study suggests that the up-regulated expression of Nav1.6 in cervical cancer could be useful as a new marker for the metastatic behavior of this tumor. Additionally, Na1.6 expression is significantly up-regulated during transformation of cervical epithelium increasing MMP-2 activity and causing metastatic invasiveness of cervical cancer cells (36).

#### Colon Cancer

Using RT-PCR and immunohistochemistry the expression of Nav1.5 has been detected in HT29, SW620, and SW480 colon cancer cells. Furthermore, strong Nav1.5 protein staining was found in colon cancer specimens while little to no staining was detected in matching normal colon tissues. Importantly, the invasive potential of colon cancer cells was inhibited by Nav1.5-specific small interfering RNAs, indicating that the Nav1.5-encoding gene *SCN5A* may be involved in the regulation of cell invasion-related signaling pathways (7). Recently, the same group could associate channel activity with Rap1-dependent sustained MAPK activation in the human colon cancer cell line SW620 by using pharmacological inhibitors/activators and siRNA interference. And they further demonstrated that activation of sodium channel-induced expression changes of invasion-related genes through the PKA/ERK/c-JUN/ELK-1/ETS-1 transcriptional pathway (37).

#### Lung Cancer

Lung cancer is histopathologically divided into two major categories: non-small cell lung cancer and small cell lung cancer which are accounting for 80 and 20% of lung cancer, respectively. In the human small-cell lung cancer (SCLC) cell line H510, Nav1.3, 1.5, 1.6, 1.9 are expressed and may enhance the capacity of tumor cells for endocytosis suggesting that a high expression of VGSC could be responsible for the increased capacity of tumor cells to metastasize (38). In human non-small cell lung cancer, voltage-dependent sodium currents are present in strongly metastatic H23 and H460 cells, whereas no VGSC current has been found in normal NL20 cells or in low metastatic A549 cells. The VGSC blocker TTX reduces the invasive capacity of H23, H460 cells by 40–50% while it has no effect in A549, NL-20, and BEAS-2B cells (39). Together, these results suggest that functional expression of VGSC might be an integral component of the metastatic process in lung cancer cells.

#### Ovarian Cancer

The relative mRNA expression levels of Nav1.1, Nav1.3, Nav1.4, and Nav1.5 in ovarian cancer cells are significantly higher than those in normal ovarian tissues (5). Furthermore, the mRNA expression levels of Nav1.2, Nav1.4, Nav1.5, and Nav1.7 are greatly increased in the strongly metastatic ovarian cancer cell lines, Caov-3 and SKOV-3, compared to the weakly metastatic Anglne cells. In addition, treatment of Caov-3 and SKOV-3 cells with TTX reduces their migration and invasion by 50–60% with no effect on proliferation. Immunohistochemical staining and Western blot results show that the Nav1.5 protein is significantly elevated in ovarian cancer tissue and cell lines compared to normal ovarian tissues suggesting that *SCN5A*/Nav1.5 is important for occurrence and development of ovarian cancer.

### The Mechanism of VGSC Regulating Cancer Metastasis

**Figure 2:**
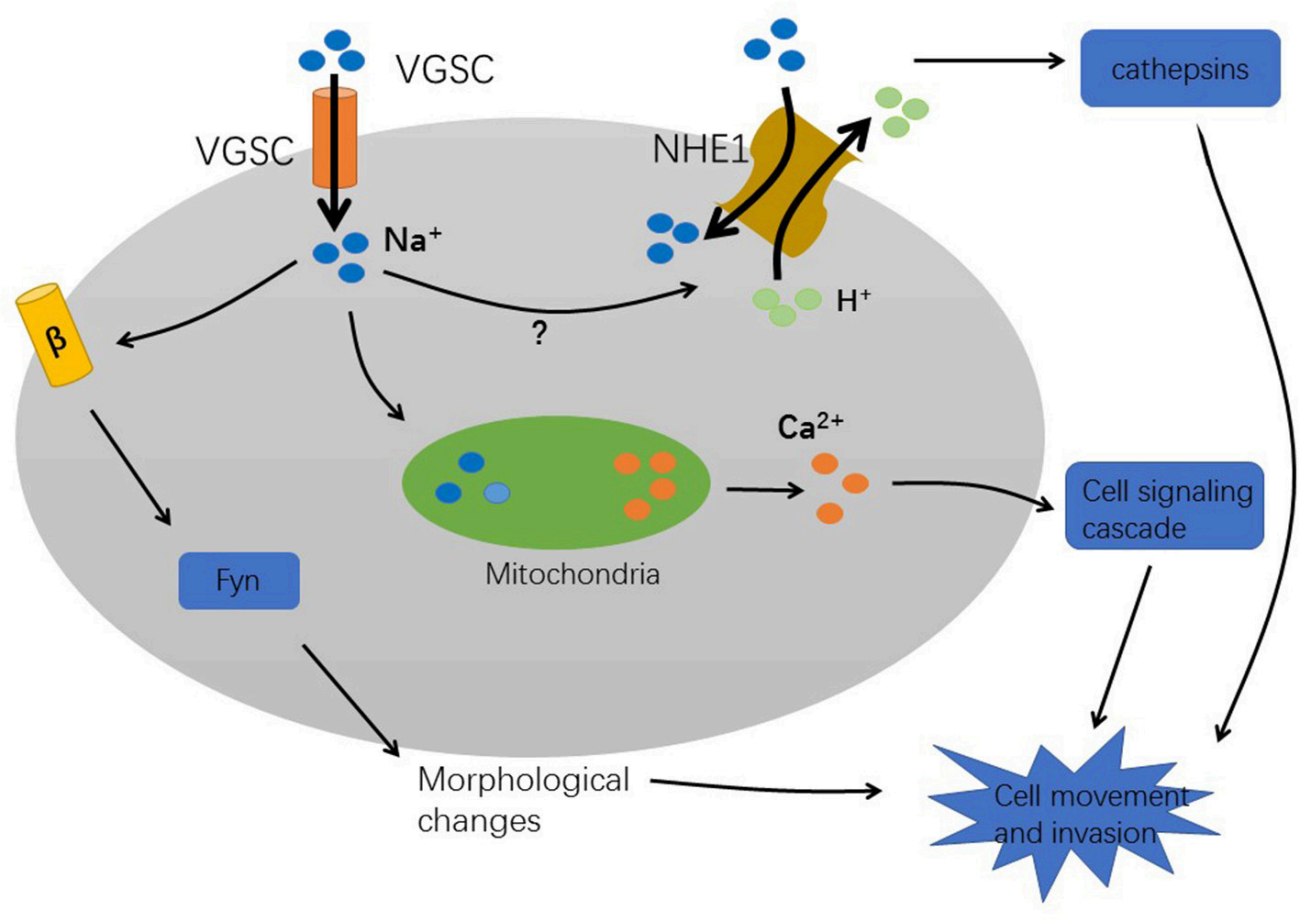
The mechanism of VGSC-induced cancer metastasis.

Numerous studies have demonstrated that VGSC plays an important role in tumor metastasis. However, the mechanism by which VGSC promotes the metastasis of cancer cells is not yet fully understood. Currently, there are some hypotheses (Figure 2).

Firstly, sodium influx could be involved in tumor progression. The high expression of VGSC in tumors causes strong Na+ influxes. The Sodium–hydrogen antiporter 1 (NHE1), a hydrogen ion channel is co-expressed with VGSC to regulate H^+^ efflux. This leads to intracellular alkalization and extracellular acidification. An extracellular acidic pH facilitates the activation of cysteine cathepsins B and S that degrade the extracellular matrix, thereby enhancing pH-dependent tumor cell invasion and metastasis (30, 40, 41). Furthermore, study in MDA-MB-231 breast cancer cells indicating that Nav1.5 expression increases Src kinase activity and the phosphorylation of the actin-nucleation promoting factor cortactin. This results in cytoskeletal changes such as the formation of invadopodia. Subsequently, the cells acquire an elongated, invasive morphology which increases the ability of cells to metastasize (42). Furthermore, Na^+^ influx activates voltage-gated calcium channels, which increase intracellular Ca^2+^ concentration. After VGSC activation, mitochondria rapidly uptake Na^+^ and then release Ca^2+^ into the cytosol (32). This process enhances the formation of podosomes/invadopodia and promotes tumor cell invasion (43). However, it is unclear how VGSCs which are present in the vesicle membrane interact with VGSCs present in the plasma membrane. A mechanism that eludes to a role of VGSC in non-excitable tumor cells has been proposed in an analysis of these channels in astrocytes. In these glia cells the presence of VGSC could provide a pathway for sodium ions to return to the cytoplasm and thus to maintain the activity of the Na+/K(+)-ATPase (44). This mechanism is proposed to contribute to the ion homeostasis in the extracellular space in the brain and could also be important in tumors.

Secondly, the behavior of VGSCs that alters tumor cells to a metastatic phenotype is related to the β subunit. It has been reported that in breast cancer cells, β1 mRNA and protein levels are significantly higher in the weak metastatic MCF-7 cell line than in the high metastatic MDA-MB-231 cell line. Inhibition of β1 expression in MCF-7 cells by siRNA increased cell motility by reducing cell adhesion (45). In addition, overexpression of β2 in prostate cancer LNCaP cells increases cell migration and invasion (46). Therefore, β1 and β2 may play different functional roles in different cells, and the tumor *in vivo* may be different compared with cultured cells. However, since the β subunits are Ig family cell adhesion molecules, it can be speculated that their regulation of cancer cell metastatic behavior may be through regulation of adhesion/separation. Moreover, fyn kinase is a critical signaling intermediary in the mechanism during β1-mediated process outgrowth in tumor cells (47). Recently, in the study of breast cancer, it was first demonstrated that *SCN4B* /β4 protein is expressed in non-cancer epithelial cells, but expression was reduced in cancer tissues, and was almost absent in high-grade tumors and metastases. In cancer cells, by promoting the amoeboid-mesenchymal hybrid phenotype, reducing β4 expression increases RhoA activity and enhances cell migration and invasiveness. In contrast, overexpression of *SCN4B*/β4 reduced the aggressiveness of cancer cells, tumor growth, and metastasis progression (48). Similarly, a retrospective study of the database revealed that mRNA and protein levels of *SCN4B* were significantly down-regulated in papillary thyroid cancer (PTC) compared to normal thyroid tissue, and its expression may be inhibited by DNA hypermethylation (49). These findings indicate that *SCN4B*/β4 may be a suppressor gene of cancer metastasis.

Moreover, VGSC can regulate gene expression. Currently, a novel gene interaction network related to cancer invasion has been simulated in HT29 colon cancer cell line and *SCN5A* is a key regulator of the gene networks involved in metastatic invasion (7). This suggests that Nav1.5 may be an early entry point to the signaling mechanism.

The sodium ion influx activates the Sodium–hydrogen antiporter NHE1, which leads to a low intracellular pH, enhances the hydrolysis activity of cathepsins, and degrades the extracellular matrix. In addition, after VGSC activation, mitochondria rapidly absorb Na^+^ and release Ca^2+^, and then activate a cell-signaling cascade to promote the formation of invasive podosomes. These processes enhance the cell invasion ability. The β subunit changes the cell morphology and affects the movement of cells through adhesion mechanisms that require fyn kinase and Na^+^ current.

### Molecular Mechanisms of VGSC Regulation

Various molecular mechanisms regulate VGSC expression and function including the presence of growth factors and the effects of hormones. Additionally, the VGSC expression has been shown to have a negative feedback effect on its own expression.

#### Growth Factors

Growth factors play an important role in the occurrence and development of tumors. Their receptors and corresponding signaling pathways are considered to be putative targets of tumor therapy (50, 51). In prostate and non-small cell lung cancer cells, epidermal growth factor (EGF) can upregulate the expression of Nav1.7 and promote the migration of cancer cells (52, 53) or promote cell invasion through the ERK1 /2 pathway, respectively (54). The study of non-small cell lung cancer shows that the ability of EGF to promote cell invasion deceases after silencing Nav1.7 expression, suggesting that EGF can enhance cell invasion by regulating VGSC (48). Furthermore, vascular endothelial growth factor (VEGF) can promote angiogenesis that plays an important role in the process of tumor cell invasion (55). Currently, in the DRG neurons of the bladder, VEGF has been found to increase cellular excitability by up-regulating VGSC expression (56). In addition, stimulation of mouse prostate cancer cells with nerve growth factor (NGF) upregulates the expression of Nav1.7, and in human prostate cancer cells, the promoter region of *SCN9A* (encoding Nav1.7) can be activated by NGF (57, 58).

#### Hormones

A study found that in cardiomyocytes, Foxo1 directly binds the insulin response elements in the *SCN5A* promoter region and negatively regulates Nav1.5 expression (59). In adrenal cells, insulin upregulated the expression of Nav1.7, which was mediated by PI3K-induced GSK-3β inhibition (60). In addition, in MDA-MB-231 breast cancer cells, insulin can upregulate VGSC expression in the plasma membrane and enhance the ability of cell migration (61). In human BCa cells, strongly metastatic MDA-MB-231 cells express functional VGSC but lack ER. Conversely, weakly metastatic MCF-7 cells are ER positive and do not express any functional VGSC (31). Similarly, there is a negative association between basal expression of androgen receptor (AR) and VGSC in PCa cells, i.e., weakly/non-metastatic cells do not possess functional VGSCs (62). However, the latest studies in patients with non-metastatic colon cancer show that high expression of Nav1.5 is associated with high ER-β expression and has been identified as a predictor of low 5-year DFS rates in patients (63). In conclusion, the expression of VGSC is closely associated with the secretion of hormones.

#### Self-Regulation

In excitable cells, the expression of VGSCs is often regulated by negative feedback. Chronic treatment with VGSC openers leads to a reduction of mRNA/protein expression, conversely, treatment with VGSC blockers increases functional protein expression (64–66). However, there is positive feedback in the expression of Nav1.7 in strongly metastatic PCa MAT-LyLu cells. After TTX inhibition of VGSC, phosphorylation of PKA was reduced. At the same time, after the use of PKA inhibitors, voltage-gated sodium ion current decreased, while agonists added with PKA increased voltage-gated sodium currents (67, 68). A similar positive feedback mechanism can be found in MDA-MB-231 breast cancer cells (69).

## Concluding Remarks

Malignant tumors constitute a serious threat to human health. With the deepening of interdisciplinary research in molecular biology, cell biology, and pharmacology, the research on the relationship between ion channels and tumors has made great progress. It has been elucidated that VGSCs can be expressed in invasive cancer cells and can increase the ability of tumor cells to move and invade. Therefore, they can be considered to be important regulators of cancer development. However, the expression of the α and β subunits of VGSCs in different tumors and their role in disease progression need to be further investigated. In addition, the molecular mechanisms involved in the regulation of VGSCs activity are still unclear. Some of the channel blockers currently being developed may act as an intervention for metastatic disease. This will facilitate the use of VGSCs as a diagnostic marker for early diagnosis and as a therapeutic target in the treatment of clinical metastatic tumor diseases.

## Author Contributions

WM and JZ wrote the manuscript and designed the figures. SY, YJ, and HK corrected and supervised the manuscript.

### Conflict of Interest Statement

The authors declare that the research was conducted in the absence of any commercial or financial relationships that could be construed as a potential conflict of interest.
